# Comparison of Endoscopic Ultrasound‐guided Tissue Acquisition and Percutaneous Liver Biopsy for Diagnosing Focal Liver Lesions: A Retrospective Single‐Center Study

**DOI:** 10.1002/deo2.70224

**Published:** 2025-10-15

**Authors:** Kei Yane, Keita Seto, Yuki Ikeda, Kotaro Morita, Mayu Shimizu, Koki Yoshida, Sota Hirokawa, Tetsuya Sumiyoshi, Michiaki Hirayama, Hitoshi Kondo

**Affiliations:** ^1^ Department of Gastroenterology Tonan Hospital Sapporo Japan

**Keywords:** endosonography, EUS‐TA, interventional ultrasonography, liver neoplasms, needle biopsy

## Abstract

**Background and Aim:**

Accurate diagnosis of focal liver lesions (FLLs) is crucial for determining the appropriate treatment strategies. Although percutaneous liver biopsy (PLB) is the standard diagnostic procedure, it has limitations, particularly for difficult‐to‐access lesions. Endoscopic ultrasound‐guided tissue acquisition (EUS‐TA) is an emerging diagnostic alternative. This study aimed to compare the diagnostic accuracy, safety, and histological adequacy of EUS‐TA with those of conventional PLB for diagnosing FLLs.

**Methods:**

This single‐center retrospective study included 70 patients who underwent EUS‐TA (*n* = 28) or PLB (*n* = 42) for FLLs between January 2019 and February 2024. Diagnostic accuracy, sensitivity, specificity, tissue adequacy, and adverse events were assessed.

**Results:**

The technical success rate was 100% in both groups. EUS‐TA showed 100% sensitivity, 100% specificity, and 100% accuracy without false negatives. PLB showed 88.6% sensitivity, 100% specificity, and 90% accuracy with four false negatives. Both methods provided sufficient specimens for immunohistochemistry. Adverse events were rare. However, postprocedural pain (7.1%) and 1 case of needle tract seeding (2.4%) occurred in the PLB group, whereas one bleeding event (3.6%) occurred in the EUS‐TA group. EUS‐TA was mainly used for left and caudate lobe lesions.

**Conclusions:**

EUS‐TA appears to be a safe and accurate option for FLLs, particularly when percutaneous access is difficult. In clinical practice, EUS‐TA and PLB may serve complementary roles, and their combined use could help improve diagnostic accuracy. Prospective studies are needed to clarify specific indications.

## 1 Introduction

Focal liver lesions (FLLs) encompass primary hepatic malignancies, metastatic tumors, and various non‐neoplastic conditions. Their accurate histopathological diagnosis is crucial for determining appropriate treatment [1, 2]. Despite advancements in non‐invasive imaging, histological confirmation remains necessary for many FLLs, particularly when imaging cannot provide a definitive diagnosis or in patients with multiple cancers, including prior malignancies.

Traditionally, ultrasound (US)‐ or computed tomography (CT)‐guided percutaneous liver biopsy (PLB) has been widely used for the histological diagnosis of FLLs [2, 3]. Although PLB provides valuable diagnostic information, limitations have been reported [4], such as difficulty in puncturing lesions depending on their location.

Recently, endoscopic US‐guided tissue acquisition (EUS‐TA) for FLLs has become a promising approach to overcome these challenges [5, 6, 7, 8, 9, 10, 11, 12, 13]. EUS‐TA involves detailed visualization of the liver and hepatic lesions from the gastrointestinal lumen using high‐frequency ultrasound, followed by real‐time guided tissue acquisition. EUS‐TA is anticipated to facilitate access to lesions located in areas difficult to reach percutaneously and enable safer tissue sampling. EUS‐TA for FLLs has a diagnostic accuracy of 89.7% to 100%, with an adverse event rate of 1%–3% [14, 15, 16, 17, 18]. However, studies directly comparing the efficacy and safety of EUS‐TA with conventional PLB for FLLs remain limited [19, 20]. The present study aimed to compare the diagnostic accuracy, safety, and histological adequacy of EUS‐TA with those of conventional PLB for diagnosing FLLs.

## 2 Materials and Methods

We used a single‐center retrospective cohort study. We included patients who underwent EUS‐TA or US‐guided PLB for FLLs at Tonan Hospital between January 1, 2019 and February 29, 2024. Clinical data were extracted from electronic medical records and endoscopy databases, and analyses were conducted based on medical records, pathology reports, laboratory findings, and radiological assessments.

Eligible lesions included primary liver malignancies, metastatic liver tumors, benign hepatic masses, and undetermined lesions requiring histological diagnosis. Adult patients 18 years or older were eligible for inclusion if they had CT‐, magnetic resonance imaging‐, or US‐confirmed FLL, and subsequently underwent EUS‐TA or PLB. The exclusion criteria comprised coagulopathy (international normalized ratio [INR] > 1.5 or platelet count < 50,000/mm^3^). We excluded cases of biopsy on patients with an established pathological diagnosis solely to obtain a sample for cancer genomic profiling.

### 2.1 EUS‐TA and PLB Procedures

#### 2.1.1 EUS‐TA Procedure

**FIGURE 1:**
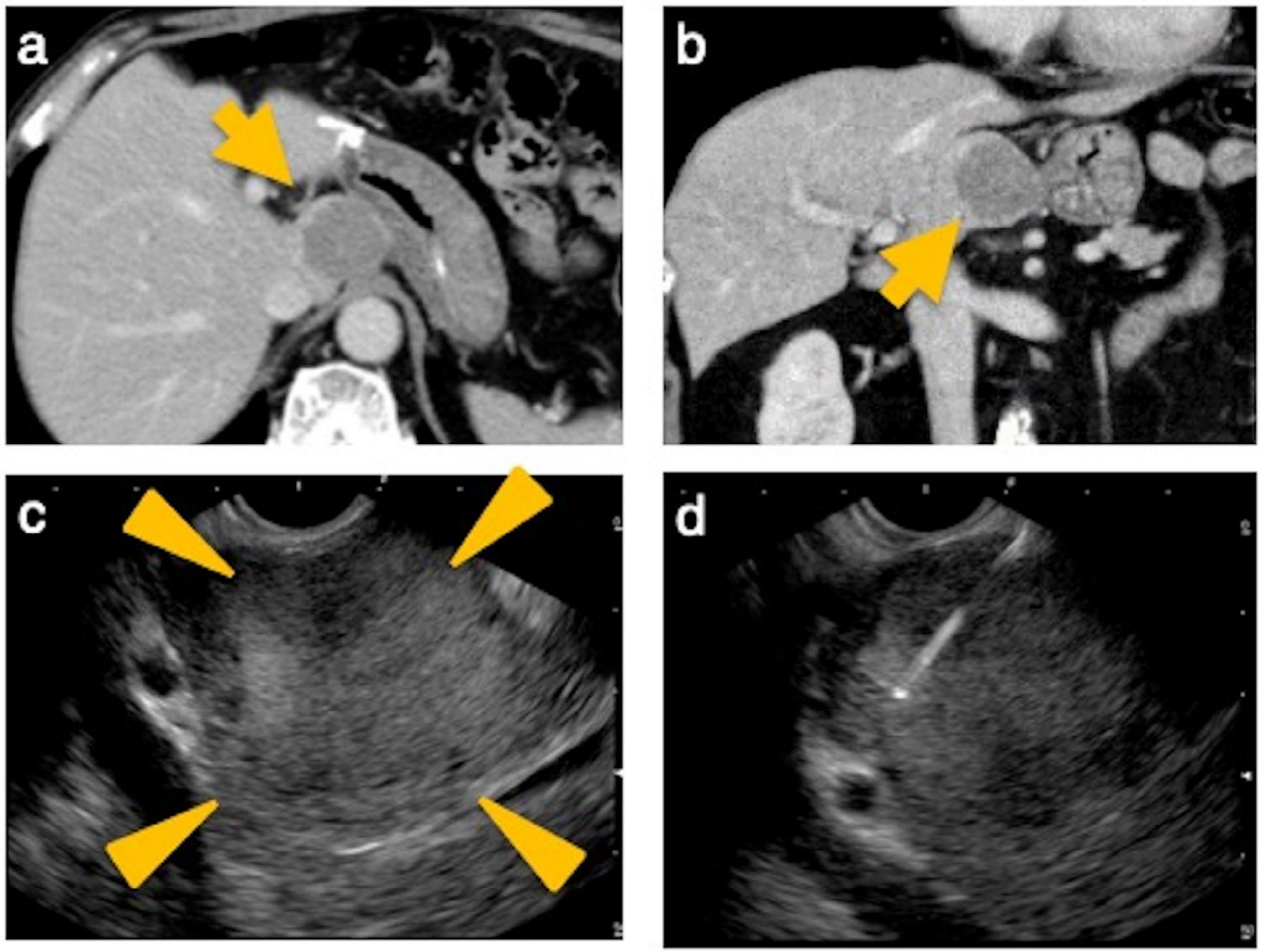
Endoscopic ultrasound‐guided tissue acquisition (EUS‐TA) for a caudate lobe mass. (a) Computed tomography (CT) image (axial). A 30‐mm low‐attenuation mass is observed in the caudate lobe. (b) CT image (coronal). The lesion is located adjacent to the stomach. (c) Endoscopic ultrasound (EUS) image. The lesion was easily visualized. (d) EUS‐TA was performed. The lesion was diagnosed as a metastatic liver tumor.

EUS‐TA was performed or supervised by an experienced endoscopist (Kei Yane, >500 EUS‐TA procedures performed) using linear‐array echoendoscopes (EG580UT or EG740UT; Fujifilm Corporation, Tokyo, Japan) (Figure 1). Pentazocine and midazolam were administered, and the procedure was performed under conscious sedation. The puncture route was selected based on the lesion location. A 19‐ or 22‐G fine‐needle biopsy (FNB) needle (Acquire; Boston Scientific, Natick, MA, USA) was used. The standard number of needle passes was two. Suction was applied using a 20 mL syringe. Rapid on‐site evaluation (ROSE) was performed in all cases. If ROSE indicated an inadequate sample, additional passes were performed.

#### 2.1.2 PLB Procedure

US‐guided PLB was performed or supervised by trained gastroenterologists or radiologists using an 18‐G automatic Tru‐Cut needle (FINECORE; Toray Industries Inc., Tokyo, Japan). Patients underwent PLB under local anesthesia. The lesion was visualized by transabdominal ultrasound. Color Doppler was used to ensure that no major vessels were in the planned puncture route. After a local anesthesia to the skin, a small skin incision was made with a scalpel, and subcutaneous tissue was bluntly dissected using mosquito forceps before needle insertion. Two passes were performed. If the sample was visually insufficient, additional passes were performed.

#### 2.1.3 Post‐Procedure Care (Common to Both EUS‐TA and PLB Groups)

Following EUS‐TA and PLB, the patients in both groups were instructed to rest for 2 h. The following day, the patients underwent blood tests and were assessed for abdominal symptoms and other complications.

### 2.2 Outcome Measures

The primary outcome was diagnostic accuracy, defined as concordance between the biopsy diagnosis and the final diagnosis. If a biopsy indicated malignancy (positive or suspicious) and was consistent with the clinical course, it was considered a correct diagnosis (true positive). If a biopsy indicated benignity, it was considered a correct diagnosis if the lesion showed no change, regressed, or resolved over a minimum of 6 months of follow‐up (true negative). If a biopsy indicated benignity but the lesion subsequently progressed or enlarged, or if malignancy was confirmed by another biopsy method, it was classified as an incorrect diagnosis (false negative). Sensitivity, specificity, and accuracy were calculated based on the differentiation between benign and malignant lesions. The secondary outcomes included histological adequacy (particularly for immunohistochemistry), type of biopsy needle used, number of needle passes, and incidence of adverse events (bleeding, infection, postprocedural pain). Adverse events were defined based on the American Society for Gastrointestinal Endoscopy lexicon [21]. Postprocedural pain was defined as new‐onset pain occurring within 24 h post‐procedure that required analgesics.

### 2.3 Statistical Analysis

All statistical analyses were performed using EZR [22]. Continuous variables were presented as means ± standard deviation (SD) or medians (range), and categorical variables as counts and percentages. The chi‐square test or Fisher's exact test was used for categorical variables, and the t‐test or Mann–Whitney U test for continuous variables. A p‐value of < 0.05 indicated a significant difference.

### 2.4 Ethical Considerations

This study was approved by the Ethics Committee of Tonan Hospital (Approval No. 1‐25‐1; March 14, 2024) and conducted in accordance with the Declaration of Helsinki. All patients received information about the procedure and provided informed consent before the biopsy. Informed consent for study participation was waived because of its retrospective design, and an opt‐out option was available to the patients.

## 3 Results

### 3.1 Patient Characteristics

We included 70 patients who underwent tissue diagnosis for FLLs between January 2019 and February 2024. Of these, 28 underwent EUS‐TA (40.0%) and 42 PLB (60.0%) (Table 1). The final diagnoses in the EUS‐TA group were 26 malignant cases.

**TABLE 1:** Patient characteristics.

	EUS‐TA group (*n* = 28)	PLB group (*n* = 42^#^)	*p*‐Value
Age, median (range), years	73 (51–94)	74.5 (50–89)	0.98^*^
Sex (male/female)	17/11	24/18	0.81^**^
Reason for selecting EUS‐TA			
Simultaneous evaluation with other sites	50.0% (14/28)		
Difficulty in performing percutaneous puncture	21.4% (6/28)		
Others	28.6% (8/28)		
Malignant disease, %	92.9% (26^#^/28)	87.5% (35^##^/40^###^)	0.69^**^
Antithrombotic agent use, %	25% (7/28)	31% (13/42)	0.79^**^
Tumor diameter, mm	36.5 (6–180)	25 (5–120)	0.23^**^
Needle gauge	19G: 4, 22G: 24	18G: 42	
Lesion location			< 0.01^**^
Right lobe	10.7% (3/28)	81.0% (34/42)	
Left lobe	57.1% (16/28)	19.0% (8/42)	
Caudate lobe	32.1% (9/28)	0	
Puncture route			
Transgastric	89.3% (25/28)		
Transduodenal	10.7% (3/28)		
Liver parenchyma distance, mm	7 (2–30)	20 (5–65)	< 0.01^**^

Abbreviations: EUS‐TA, endoscopic ultrasound‐guided tissue acquisition; PLB, percutaneous liver biopsy.

* Mann‐Whitney U test.

** Fisher's exact test.

^#^ The final diagnoses in the EUS‐TA group were 26 malignant cases (metastatic liver tumor, *n* = 21; hepatocellular carcinoma [HCC], *n* = 3; intrahepatic cholangiocarcinoma [ICC], *n* = 1; malignant lymphoma, *n* = 1) and two benign cases (hemangioma, *n* = 1; inflammatory mass, *n* = 1).

^##^ The final diagnoses were 35 malignant cases (metastatic liver tumor, *n* = 22; HCC, *n* = 7; ICC, *n* = 4; neuroendocrine neoplasm [NEN], *n* = 1; malignant lymphoma, *n* = 1) and five benign cases (angiomyolipoma, *n* = 1; peliosis hepatis, *n* = 1; hyperplastic nodule, *n* = 1; inflammatory masses, *n* = 2).

^###^ In two cases, the biopsy results were benign, and the observation period was < 6 months.

In the PLB group, the final diagnoses were 35 malignant cases. A final diagnosis was not obtained in two cases with benign biopsy results because the follow‐up period was < 6 months. These two cases were excluded from the diagnostic accuracy analysis.

**FIGURE 2:**
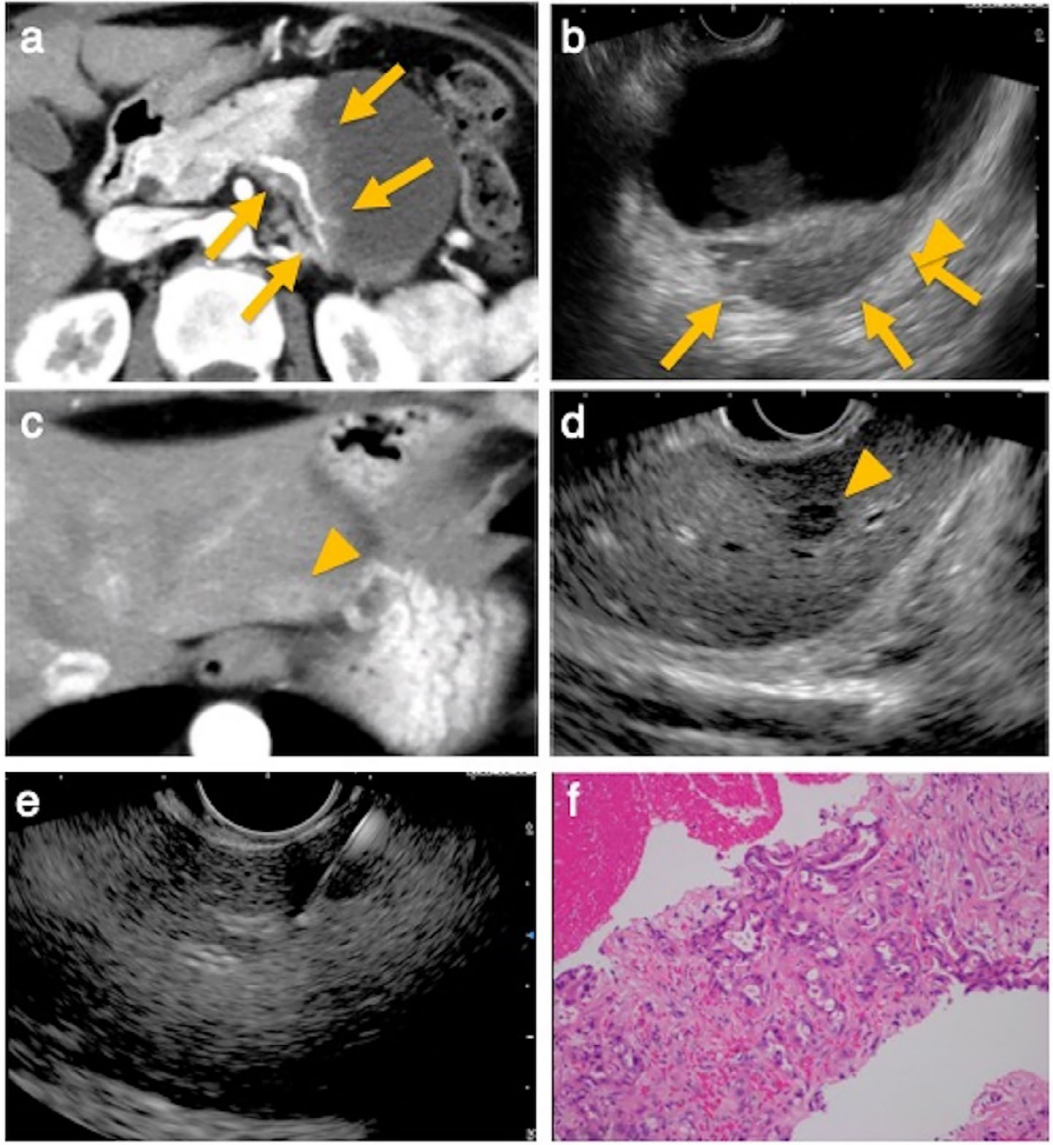
Endoscopic ultrasound‐guided tissue acquisition (EUS‐TA) for a small lesion in hepatic segment 2 (S2). (a) Computed tomography (CT) image. A low‐attenuation mass is observed in the pancreatic tail, accompanied by a large adjacent retention cyst. (b) EUS image. The lesion is visualized through the retention cyst. (c) CT image. Arterial phase contrast‐enhanced imaging reveals a ring‐enhancing lesion in segment 2 of the liver. (d) EUS image. A 6‐mm hypoechoic lesion is identified in segment 2. (e) EUS‐TA was performed. (f) Histopathological examination revealed adenocarcinoma, and the lesion was diagnosed as a metastatic liver tumor.

Before this study, PLB was our standard approach for tissue acquisition of FLLs; however, during that period, EUS‐TA was selectively used in certain cases. Of the 14 cases for simultaneous evaluation, FLL tissue acquisition was planned in seven cases. In the remaining seven cases, FLL biopsy was additionally performed during the procedure because sampling of the initially targeted lesion was difficult (Figure 2).

In the EUS‐TA group, the median age was 73 years, with 17 male patients (60.7%) and 11 female patients (39.3%). In the PLB group, the median age was 74.5 years, with 24 male patients (57.1%) and 18 female patients (42.9%). In the EUS‐TA group, 19G needles were used in four cases and 22G needles in 24 cases. In the PLB group, 18G needles were used in all cases. The lesion locations in the EUS‐TA group included the right lobe (three cases, 10.7%), left lobe (16 cases, 57.1%), and caudate lobe (nine cases, 32.1%). The case distributions according to the Couinaud classification were S1, nine cases; S2, 11 cases; S3, five cases; S5, two cases; S6, one case. Lesions in S5 and S6 in three cases were punctured transduodenally, whereas those in the remaining 25 cases were punctured transgastrically. The lesion locations in the PLB group included the right lobe (34 cases, 81.0%) and left lobe (eight cases, 19.0%); there were no caudate lobe lesions. The case distributions according to the Couinaud classification were S1, 0; S2, 0; S3, three cases; S4, five cases; S5, 17 cases; S6, seven cases; S7, one case; S8, seven cases; anterior segment, one case; posterior segment, one case. Lesion size was larger in the EUS‐TA group (median, 36.5 mm; range, 6–180 mm) than in the PLB group (median, 25 mm; range, 5–120 mm). The distance of the normal liver parenchyma traversed along the puncture route was shorter in the EUS‐TA group (median, 7 mm; range, 2–30 mm) than in the PLB group (median, 20 mm; range, 5–65 mm).

Antithrombotic agents were used in seven cases in the EUS‐TA group and 13 cases in the PLB group. In the EUS‐TA group, these medications were discontinued before the examination, with the exception of aspirin monotherapy. In the PLB group, all antithrombotic agents, including aspirin, were discontinued before the examination.

Table 1 shows the patient characteristics. Lesions in the EUS‐TA group were more frequently located in the left and caudate lobes. Lesions in the PLB group were more often found in the right lobe. A significant difference in lesion location was observed between the two groups.

### 3.2 Comparison of Diagnostic Accuracy

The technical success rate was 100% in both groups. In the EUS‐TA group, there were 26 true positives and 2 true negatives, with no false positives or false negatives (Table 2). Thus, the sensitivity, specificity, and overall accuracy were all 100%.

**TABLE 2:** Diagnostic accuracy.

	EUS‐TA group (*n* = 28)	PLB group (*n* = 42^#^)	*p*‐Value
Technical success	100% (28/28)	100% (42/42)	1^*^
Sensitivity	100% (26/26)	88.6% (31/35)	0.13^*^
Specificity	100% (2/2)	100% (5/5)	1^*^
Accuracy	100% (28/28)	90% (36/40)	0.14^*^

Abbreviations: EUS‐TA, endoscopic ultrasound‐guided tissue acquisition; PLB, percutaneous liver biopsy.

* Fisher's exact test.

^#^ In two cases, the biopsy results were benign, and the observation period was < 6 months.

In the PLB group, there were 31 true‐positives, five true‐negatives, and four false‐negatives, with no false‐positives. The 4 false negatives were finally diagnosed as three HCCs and one metastatic liver tumor of unknown primary origin. Two cases showed lesions deep in segment S8 with unclear ultrasound visualization, one case showed a lesion with internal necrosis, and the remaining case showed a relatively small lesion measuring 15 mm. The sensitivity was 88.6%, the specificity was 100%, and the accuracy was 90%. The remaining two cases were excluded from the diagnostic accuracy analysis because their biopsy results were benign and their observation period was < 6 months. Assuming both cases were correctly diagnosed, the diagnostic accuracy of the PLB group would be 90.5% (38/42). If neither were correctly diagnosed, the diagnostic accuracy would be 85.7% (36/42). Although the difference in accuracy was not significant, the absence of false‐negative cases in the EUS‐TA group is notable.

### 3.3 Secondary Outcomes

In the EUS‐TA and PLB groups, adequate specimens for histopathological examination were obtained in all cases. Immunohistochemistry was performed in 53.6% EUS‐TA cases and 59.5% PLB cases (Table 3). All yielded specimens were suitable for histological evaluation. In the remaining cases, diagnosis was possible with hematoxylin‐eosin staining.

**TABLE 3:** Secondary outcomes.

	EUS‐TA group	PLB group	*p*‐Value
	(*n* = 28)	(n = 42^#^)	
Immunohistochemistry	53.6% (15/28)	59.5% (27/42)	0.63^*^
Number of needle passes	2.07±0.81	2.43±0.80	0.07^**^
Adverse events	3.6% (1/28)	9.5% (4/42)	0.64^*^

Abbreviations: EUS‐TA, endoscopic ultrasound‐guided tissue acquisition; PLB, percutaneous liver biopsy.

* Fisher's exact test.

** Mann‐Whitney U test.

^#^ In two cases, the biopsy results were benign, and the observation period was < 6 months.

In the EUS‐TA group, a 22G needle was used in 24 cases and a 19G needle in four cases. In the PLB group, an 18‐G needle was used in all cases. The mean number of needle passes was 2.07 ± 0.81 in the EUS‐TA group and 2.43 ± 0.80 in the PLB group. Although fewer passes were needed in the EUS‐TA group, the difference was not significant.

**FIGURE 3:**
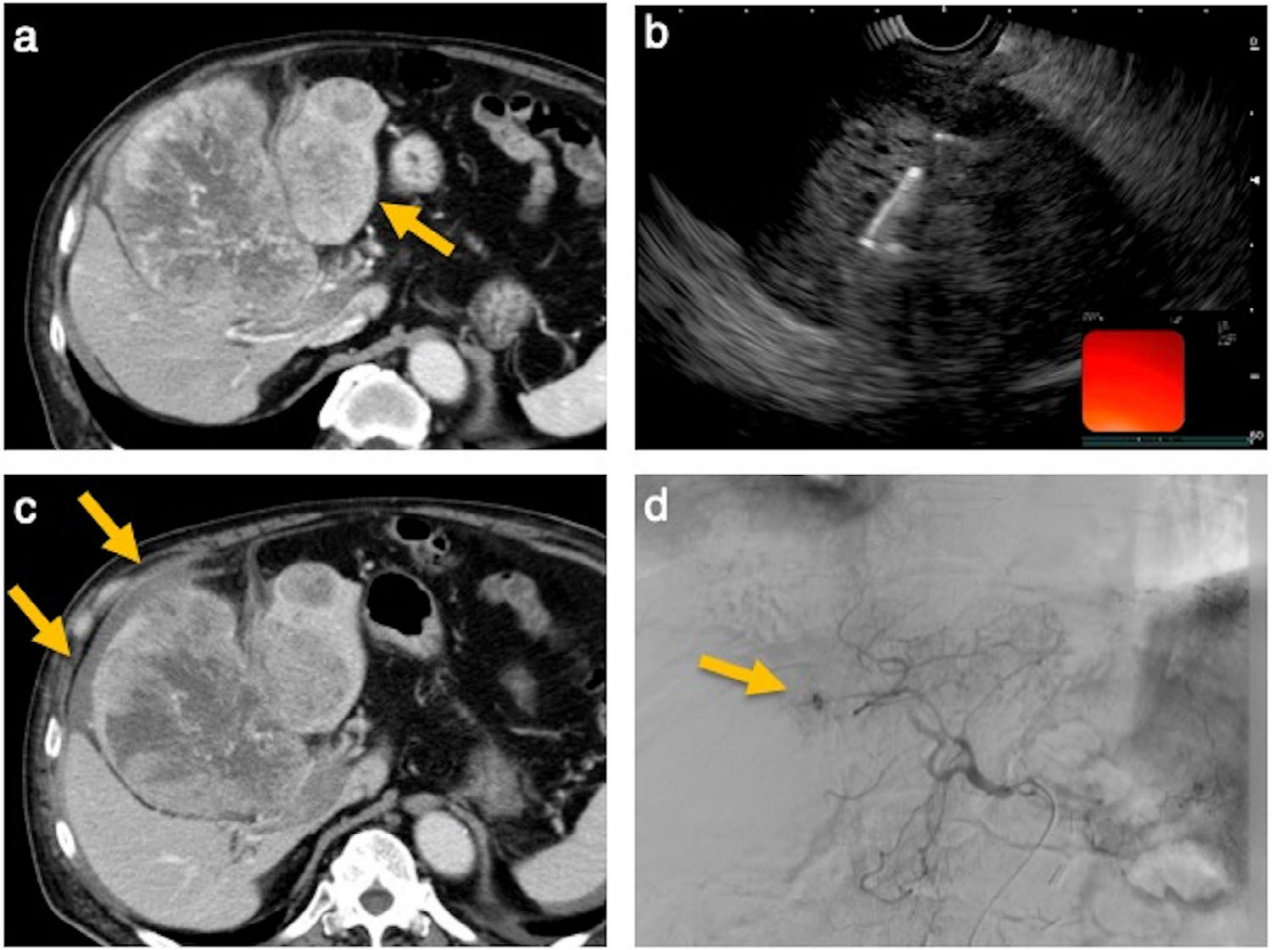
Post‐procedural bleeding following endoscopic ultrasound‐guided tissue acquisition (EUS‐TA). (a) Computed tomography (CT) image. A large mass is seen occupying the left hepatic lobe. The lesion in segment 2 is located adjacent to the stomach. (b) EUS‐TA was performed targeting the lesion in segment 2. (c) CT image 4 days after EUS‐TA. A fluid collection with higher attenuation than water is noted on the liver surface. Based on the clinical course, it was diagnosed as post‐procedural bleeding. (d) Angiographic image. Contrast extravasation from segment 4 was observed, and embolization was performed.

Adverse events were rare. One case of bleeding occurred in the EUS‐TA group (3.6%). One case of needle tract seeding (2.4%) and three cases of postprocedural pain requiring analgesics (7.1%) occurred in the PLB group. A case of bleeding was observed in the EUS‐TA group (Figure 3). Although the bleeding site did not coincide with the puncture site, a causal relationship with EUS‐TA could not be ruled out and was thus classified as a complication.

**FIGURE 4:**
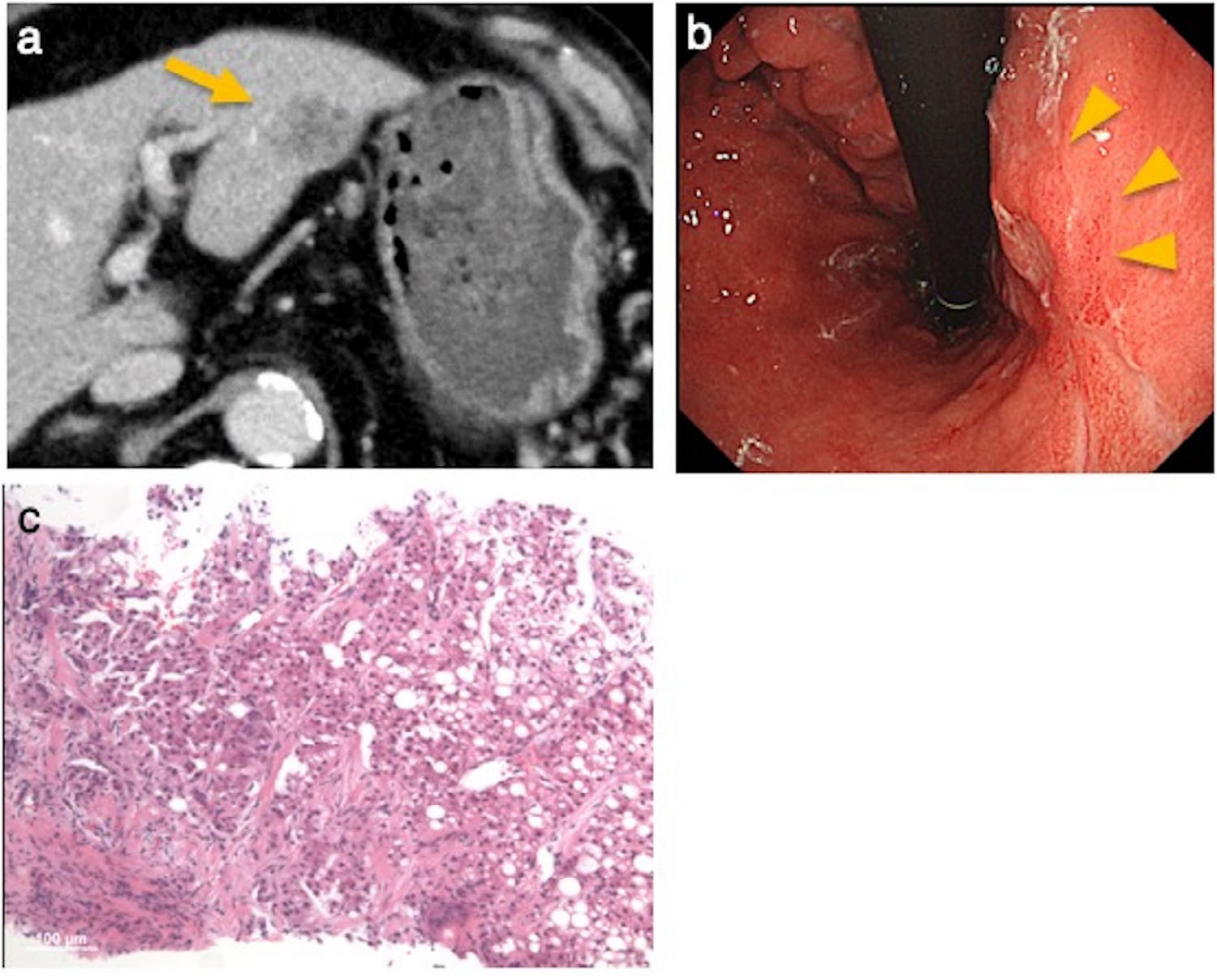
Needle tract seeding after percutaneous tumor biopsy: at the time of biopsy. (a) Computed tomography (CT) image. A low‐attenuation mass is observed in hepatic segment 3 (S3). (b) Upper gastrointestinal endoscopy. A type 3 tumor is observed on the lesser curvature of the lower gastric body. (c) Histopathological image of the liver lesion biopsy. The lesion was diagnosed as hepatocellular carcinoma.

**FIGURE 5:**
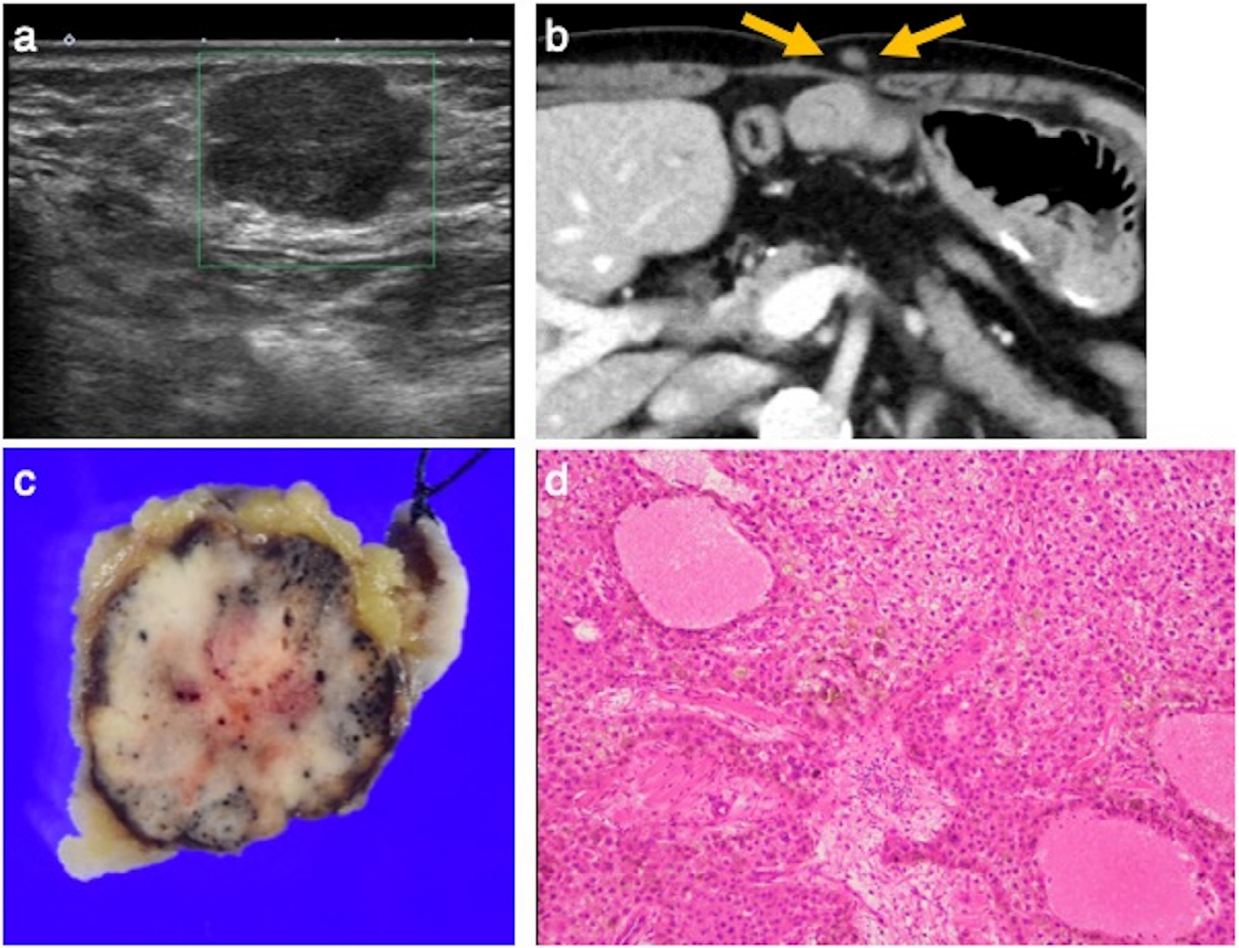
Needle tract seeding diagnosed after percutaneous tumor biopsy. (a) Ultrasound image. A hypoechoic mass is observed within the abdominal wall. (b) Computed tomography (CT) image (4 months prior to diagnosis). Retrospective review revealed a small nodule within the subcutaneous fat tissue. (c) Resected specimen. The lesion appeared as a whitish, irregularly shaped mass. (d) Histopathological image. Findings were consistent with hepatocellular carcinoma. Based on the clinical course, imaging, and pathological findings, the lesion was diagnosed as needle tract seeding following percutaneous liver biopsy.

A case of needle tract seeding in the PLB group is shown. A patient with gastric cancer and a segment 3 lesion underwent percutaneous biopsy to differentiate liver metastasis from HCC (Figure 4). Fourteen months postoperatively, a subcutaneous mass was resected, with pathology confirming HCC, consistent with needle tract seeding (Figure 5). Postprocedural pain was observed in three cases in the PLB group but not in the EUS‐TA group. This difference may be attributed to needle gauge differences and subcutaneous tissue involvement in PLB.

## 4 Discussion

We compared the diagnostic performance, safety, and histological adequacy of EUS‐TA and PLB. EUS‐TA demonstrated 100% sensitivity and specificity, with no false‐negative cases. PLB showed a slightly lower diagnostic accuracy (sensitivity: 88.6%; accuracy: 90%), although not significantly different.

Both groups achieved 100% histological adequacy for immunohistochemistry. Adverse events were infrequent; however, postprocedural pain and needle tract seeding occurred only in PLB, indicating clinical implications.

These results suggest that EUS‐TA is useful for lesions in the left lobe, particularly in segment S2, and in the caudate lobe, where PLB is difficult, as it enables puncture from adjacent sites. If tissue acquisition was insufficient after EUS‐TA for pancreatic masses or lymph nodes, additional passes can be performed immediately.

The absence of false‐negative and false‐positive results in EUS‐TA is in contrast to PLB, which had four false‐negative cases. This may reflect differences in lesion location, visibility, and technical accessibility. Two cases showed lesions located deep in segment S8 with unclear ultrasound visualization, one case showed a lesion with internal necrosis, and the remaining case showed a relatively small lesion measuring 15 mm. EUS‐TA was predominantly applied to lesions in the left and caudate lobes—areas anatomically challenging for PLB. The caudate lobe is difficult to approach percutaneously but is easily accessible via a transgastric EUS approach.

ROSE during EUS‐TA possibly contributed to the high diagnostic accuracy by enabling immediate specimen adequacy assessment [23]. Previous studies similarly reported favorable EUS‐TA results for left and caudate lobe lesions [19, 24].

Herein, the difference in sensitivity was not significant, indicating potential biases in case selection and sample size. Although complications were rare in both groups, severe adverse events such as needle tract seeding and bleeding requiring IVR were observed in two of the 70 cases. Regardless of the method, biopsy should be limited to cases in which it is essential for treatment decision‐making. Regarding postprocedural pain, analgesic‐requiring discomfort was reported exclusively in the PLB group (three cases, 7.1%); no such cases occurred in the EUS‐TA group.

This discrepancy may be attributable to the procedural route and sedation methods. PLB required penetration through the skin, subcutaneous tissue, and muscle under local anesthesia. EUS‐TA enabled direct lesion puncture from within the gastrointestinal tract under conscious sedation, possibly improving comfort. The difference in needle gauge used (18G PLB vs. predominantly 22G EUS‐TA) likely contributed to the presence or absence of pain.

The single case of needle tract seeding in PLB is a known complication associated with tumor cell dissemination. The risk of this event is theoretically lower in EUS‐TA owing to the intraluminal puncture route [25], and no such cases were observed in our study. However, our study included only a small number of cases, and theoretically, needle tract seeding can occur whenever a biopsy is performed. Therefore, large‐scale prospective validation is warranted.

Our results provide valuable clinical insights for selecting the appropriate tissue acquisition techniques for FLLs. EUS‐TA excelled in diagnostic accuracy, safety, and tissue adequacy, particularly for anatomically challenging lesions. However, PLB remains useful for right lobe lesions or cases with limited EUS access. Although some reports have demonstrated favorable EUS‐TA outcomes even for right lobe lesions, the target lesions in those studies had a median distance of only 2.3 cm from the transducer, making them relatively accessible [26]. In contrast, S7 or S8 lesions, which are difficult to visualize in close proximity by EUS, are considered technically more challenging. In our study, the right lobe lesions were limited to S5 and S6. In cases with gastrointestinal strictures or complex surgically reconstructed intestines, access to the target lesion may be restricted and may not be suitable for EUS‐TA. These modalities should therefore be viewed as complementary rather than competitive, and the selection should be tailored to lesion characteristics and patient factors. Additionally, equipment performance (EUS scopes and ultrasound processors) and the operator's technical expertise at each institution are likely to have a substantial impact. When these conditions are comparable, EUS‐TA may serve as the first choice owing to its lower associated pain. Future multicenter, prospective studies are warranted to develop standardized diagnostic strategies.

Regarding study limitations, as a retrospective single‐center analysis, selection bias and confounding factors cannot be completely excluded. The technique choice was at the physician's discretion and may have been influenced by lesion location and accessibility. Although the EUS‐TA technical success rate was 100%, this likely reflects the physicians’ selection of lesions they deemed accessible under EUS guidance based on pre‐procedural imaging, primarily CT scans. A prospective study is needed to evaluate what proportion of right lobe lesions can be successfully approached with EUS guidance. Procedural elements such as needle size, number of passes, and use of ROSE were not standardized. The sample size limited the statistical power. Nevertheless, this study provides meaningful data from a real‐world clinical setting comparing two widely used biopsy techniques.

## 5 Conclusion

EUS‐TA appears to be a safe and accurate option for FLLs, particularly when percutaneous access is difficult. In clinical practice, EUS‐TA and PLB may serve complementary roles, and their combined use could help improve diagnostic accuracy. Further large‐scale prospective studies are needed to better define indications and limitations and to support standardized diagnostic algorithms.

## Author Contributions

**Kei Yane**: conceptualization, data curation, formal analysis, investigation, visualization, writing—original draft, and writing—review & editing; **Keita Seto**: investigation; **Yuki Ikeda**: investigation; **Kotaro Morita**: investigation; **Mayu Shimizu**: investigation; **Koki Yoshida**: investigation; **Sota Hirokawa**: investigation; **Tetsuya Sumiyoshi**: supervision; **Michiaki Hirayama**: supervision; **Hitoshi Kondo**: supervision.

## Conflicts of Interest

The authors declare no conflicts of interest.

## Ethics Statement

This study was approved by the Ethics Committee of Tonan Hospital (Approval No. 1‐25‐1; approved March 14, 2024) and conducted in accordance with the Declaration of Helsinki.

## Consent

All patients provided informed consent before undergoing biopsy. The requirement for informed consent specifically for participation in this study was waived because of its retrospective design, and an opt‐out option was available to the patients.

## Clinical Trial Registration

N/A
